# An Evaluation Framework for Spectral Filter Array Cameras to Optimize Skin Diagnosis

**DOI:** 10.3390/s19214805

**Published:** 2019-11-05

**Authors:** Jacob Renzo Bauer, Jean-Baptiste Thomas, Jon Yngve Hardeberg, Rudolf M. Verdaasdonk

**Affiliations:** 1 The Norwegian Colour and Visual Computing Laboratory, Norwegian University of Science and Technology (NTNU), 2815 Gjøvik, Norway; jean.b.thomas@ntnu.no (J.-B.T.); jon.hardeberg@ntnu.no (J.Y.H.); 2 Biomedical Photonics and Imaging group, Faculty of Science and Technology, University of Twente, 7522NB Enschede, The Netherlands; r.m.verdaasdonk@utwente.nl

**Keywords:** spectral filter array, multispectral imaging, biomedical optics, image quality, reflectance spectroscopy, oxygenation, tissue optics

## Abstract

Comparing and selecting an adequate spectral filter array (SFA) camera is application-specific and usually requires extensive prior measurements. An evaluation framework for SFA cameras is proposed and three cameras are tested in the context of skin analysis. The proposed framework does not require application-specific measurements and spectral sensitivities together with the number of bands are the main focus. An optical model of skin is used to generate a specialized training set to improve spectral reconstruction. The quantitative comparison of the cameras is based on reconstruction of measured skin spectra, colorimetric accuracy, and oxygenation level estimation differences. Specific spectral sensitivity shapes influence the results directly and a 9-channel camera performed best regarding the spectral reconstruction metrics. Sensitivities at key wavelengths influence the performance of oxygenation level estimation the strongest. The proposed framework allows to compare spectral filter array cameras and can guide their application-specific development.

## 1. Introduction

Spectral filter array (SFA) cameras are a new single-shot spectral imaging technology [1], which is gaining popularity in different fields of research [2]. The light entering the camera is filtered with narrow spectral bandpass filters on each pixel or subpixel. Spatial decomposition of the spectral signal allows capturing of all spectral bands at the same instance.

Prototypes have been proposed in academia [3] and commercial models are now available including the XIMEA xiSpec camera [4,5] and Silios technologies SFA camera [6]. With increased adoption and commercial availability of SFA cameras, it is important to analyze parameters contributing to image quality parameters of these cameras and provide tools to guide further development for specific applications.

Image quality performance of cameras for close range imaging is a broad field of research [7,8,9] covering many different aspects including: spatial resolution [10,11,12], spectral or color accuracy [3,13,14], reproducibility, noise behavior [15], optical distortions and post-processing steps. The required accuracy of spectral reconstructions, number of channels and wavelength of interest are application dependent and should be evaluated in the context of specific applications. If SFAs combine accurate spectral reconstruction with real-time acquisition speed and ease of use, they could potentially be a powerful new imaging modality for the medical field. Digital imaging is already widely adopted for skin imaging, which could benefit from additional spectral information [16,17,18,19,20]. Small color variations in the skin can carry relevant information for physicians. There is a need for more reliable and quantitative methods to measure physiologic parameters of patients in non-contact. SFA cameras could combine non-contact monitoring of vital functions and diagnosis of diseased skin tissue in real time [21,22,23,24,25,26,27,28]. In particular, dynamic processes such as oxygenation would highly benefit from spectral and spatially resolved images in real time [27,29,30,31,32].

Previous work by Preece and Claridge [33] has investigated optimal filter sensitivities for a three-channel system for skin diagnosis. An extensive hardware focused analysis of spectral imagers for biomedical applications is provided by Gutiérrez-Gutiérez et al. [34]. The main focus of their work was the technical limitations including acquisition speed, efficiency, object plane curvature, spatial resolution, distortions, and noise. They emphasized an imaging system for biomedical applications should be selected after thorough testing of these parameters. A comprehensive emulation framework has been proposed by Saager et al. [35] giving an overview of the performance of different spectral imagers including a Xispec SFA camera and an RGB sensor a burn wound mouse model and photoaging experiment. High-resolution spectral measurements were performed using a spatial frequency domain spectroscopy (SFDS) system. In the computer graphic domain with Jimenez et al. [36] and Iglesias-Guitian et al. [37] described physically based skin appearance models to show color changes due to emotions or ageing. The same models can be used as to generate skin reflectance training sets.

The aim of this study is the development and testing of a framework for comparison of SFA cameras for spectral reconstruction, skin imaging, and oxygenation level estimation without prior patient measurements. A generated specialized training set is quantified for spectral reconstruction.

This framework could be considered prior to the hardware focused selection by Gutiérrez-Gutiérez et al. [34] and provides a simplified measurement free alternative to the method proposed by Saager et al. [35]. The framework could also be applied as a guide for the development of application-specific SFA cameras.

Three aims of study can be formulated as:

- comparison framework of spectral filter array cameras for skin imaging and medical diagnosis
- illustrate the impact of spectral reflectance reconstruction using a specialized training set for SFA camera applications in skin imaging.
- recommendation of commercially available SFA cameras for monitoring of vital functions and diagnosis.

## 2. The Proposed Framework

**Figure 1:**
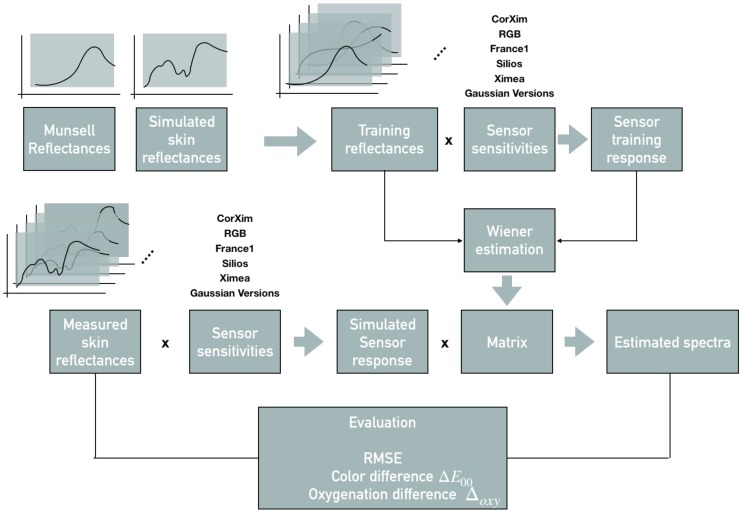
Framework, including training set, testing set, sensor sensitivities, reconstructed spectra and the evaluation according to RMSE, $\Delta E_{00}$, ΔOxy.

The proposed framework has three main elements: (1) calculation of a spectral reconstruction matrix, (2) simulated sensor responses and (3) an evaluation block. It is shown in Figure 1 and follows the concepts of a spectral filter array processing pipeline proposed by Lapray et al. [38].

As a first part, a spectral reconstruction is performed to estimate the full spectra using the limited number of SFA bands providing a measure of the performance of the different cameras independent of applications. In addition, the estimated spectra are then analyzed regarding their accuracy for oxygenation level estimation being an example for a specific application. Three SFA cameras, one prototypical, two commercially available and an RGB camera are evaluated. The impact of gaussian spectral bands (GSB) is tested by simulating sensor sensitivities with gaussian shapes for each of the SFA cameras channels.

A set of (10,000) [39] skin reflectances is generated using a Monte Carlo skin model and compared to a Munsell reflectance patch database [40,41] for training the spectral reconstruction. A database of spectral measurements of skin reflectances (100 measurements) [42] is used for testing the spectral reflectance reconstruction. The spectral reconstruction accuracy is compared numerically using Root Mean Square Error (RMSE) and $\Delta E_{00}$ color differences [43]. Differences in estimated oxygenation levels are numerically compared using a proposed metric. Spatial aspects are not considered in this study since the standard clinical measurement of oxygenation levels are usually averaged over a small area and the skin simulation is only considering homogeneous tissue over the simulated surface.

## 3. Prerequisites

For full spectral reconstruction simulated sensor responses are needed. The spectral reconstruction accuracy needs to be evaluated regarding spectral accuracy and in relation to specific applications. The framework could be applied to any channel-based spectral imager with known spectral sensitivities. For comparing specific spectral imagers, sensor sensitivities, training and test data and evaluation metrics must be chosen.

### 3.1. Spectral Imaging Model and Spectral Reconstruction

Spectral reconstruction is a useful estimation technique to estimate full spectra from a limited number of bands. The wavelengths of interest might also be unknown prior to the practical applications. It allows comparison of spectral cameras with different sensitivity peaks in a common space.

The spectral reconstruction is based on the inversion of a commonly known imaging model, which can be described with the equation:

(1) $$P_{i}=\int_{\lambda }^{}E\left(\lambda \right)R_{j}\left(\lambda \right)Q_{i}\left(\lambda \right)d\lambda$$

where $P_{i}$ is the channel response of the $i^{th}$ channel of the sensor. E(λ) is the illumination spectral power distribution (SPD) per wavelength, $R_{j}\left(\lambda \right)$ is the spectral reflectance of sample *j* and $Q_{i}\left(\lambda \right)$ describes the spectral sensitivity of the $i^{}$th channel of the sensor. Noise can be described as an additive constant to each channel.

Two simplification have been applied to the imaging model for this study. Noise per channel has not been considered and illumination has been assumed to be of equi-energy. Both variables influence the performance of the cameras in a real setup. Specific light-source power distributions might favor a particular camera hindering the comparability. A mathematical description of noise might not be an adequate descriptions of practical noise behavior of a physical camera. A chosen noise model could also favor one camera for the comparison.

This model can be inverted for spectral reconstruction, by estimating $R_{j}\left(\lambda \right)$. Several different techniques have been proposed including the pseudo-inverse method [44] (linear least-square fitting) or linear least-square fitting in lower-dimensional space (Imai–Berns method) [45]. For this study, a commonly used spectral reconstruction technique known as Wiener estimation [45,46,47,48,49,50] is applied. Before inverting Equation (1) it is rewritten into discrete formulation:

(2) $$P_{i}=\sum_{k=0}^{N}E\left(\lambda_{k}\right)R_{j}\left(\lambda_{k}\right)Q_{i}\left(\lambda_{k}\right)$$

*N* is the number of spectral bands depending on the wavelength range and spectral resolution, in this case, λ∈[400,700] with a sampling rate of 2 nm steps and N=151. For all *j* reflectances of the training set, the channels *i* of the sensor and *k* distinct spectral bands, we can write in matrix form:

(3) p=REQ

p is of J×I dimensionality with *J* spectral samples and *I* channels, R of J×N, E of N×N (diagonal matrix) and Q of N×I where *N* is 151 different wavelengths for this research. This is inverted according to the Wiener estimation method [45,46,47], in this study the implementation by Nishidate et al. [49] is followed and describes a reconstructed reflectance with:

(4) $$\tilde{r}=\mathrm{Wp},$$

where W describes the Wiener estimation matrix, $\tilde{r}$ the resulting vector of reflectance estimation or reconstruction and p the vector of sensor responses for each channel. The Wiener matrix is calculated by minimizing the square error of reconstructed and given reflectance for a training set of reflectances.

This matrix needs to be calculated for each camera and training set combination. Sensor responses can be simulated by multiplying the sensor sensitivities and the reflectance spectrum of an object. Spectral reconstructions can then be performed given this sensor response and the pre-trained Wiener estimation matrix W.

### 3.2. Sensors

Most SFA sensors are based on micro interference filters (often Fabry–Pérot interference) that can be simulated with GSB as shown by Lapray et al. [51] with width and shape as main parameters [52,53].

The framework enables the comparison of any multi-band sensors with known spectral sensitivities or optimize the design of ’virtual’ SFA cameras for specific applications. SFA cameras have a limited number of wavelength bands divided over the sensor. The design of SFA sensors will be a trade-off between spectral resolution and spectral range covered. A narrower spectral band per filter will improve the spectral resolution, but would require more spectral bands to cover the whole sensitivity. Broader sensitivities on the other hand, reduce the spectral resolution, but require less filters and avoid (“holes”) in the covered spectrum. However, for specific applications only a few primary wavelengths are needed as in case of oxygenation estimation.

In this study, we included simulated GSB they were chosen with a full width half max that make them comparable with them real sensor sensitivities of the cameras tested.

### 3.3. Training and Test Set

The training set will contribute to the accuracy of spectral reconstruction using Wiener estimation which calculates a transformation matrix that translates SFA responses to a full spectrum. This transformation matrix should minimize the difference between the reference spectrum and a reconstructed spectrum. The reference spectrum used to determine this matrix is called the training set.

For training two sets were compared to see the impact on the reconstruction accuracy for the different cameras: The Munsell database is used as a standard for color testing and the second training set was a generated for skin color simulation using a wide array of skin optical properties. The skin simulation (training set) assumes an equi-energy illumination and therefore represents illumination corrected skin spectra. Both sets are normalized using a feature scaling so that all values cover a range from 0 to 1. A more detailed description of this skin database follows in the experimental setup. For the validation if the spectral reconstruction another set based on skin reflectances was used. These skin reflectances (test set) are measured using a spectrophotometer and illumination corrected as described in [42].

**Figure 2:**
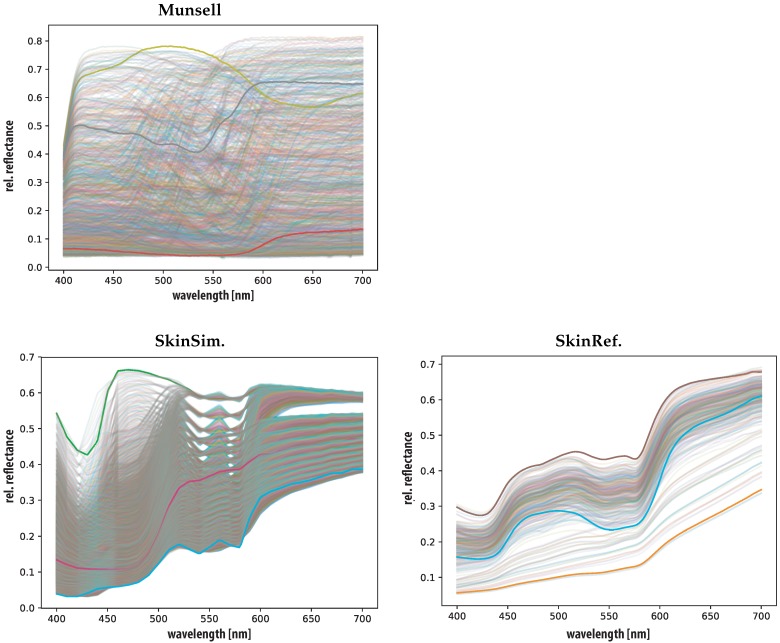
Measured Munsell relfectances [40] (Munsell), measured skin reflectances [42] (SkinRef), simulated skin reflectances (SkinSim). Three reflectances highlighted for visibility in each set.

The three sets are illustrated in Figure 2. This Figure allows comparison of the area covered by all sets and highlights three reflectances for each dataset. It includes the database of 100 measured skin reflectances [42], 10000 Monte Carlo simulated reflectances and the Munsell reflectances color patches [40,41].

### 3.4. Evaluation Metrics

The validation of the proposed framework can be tested by applying it to a specific clinical application, oxygen level estimation. This should show which spectral filter array camera is most suitable for this specific application. Three different evaluation metrics are considered. Two of the metrics focus on spectral reconstruction quality regarding shape and color. The third metric is application-specific and in this case quantifies the ability of each camera to estimate oxygen levels, it will be discussed in detail in the next section.

The first metric calculates the color difference $\Delta E_{00}$ [43] of two spectra which is the distance between two colors in the human perceptual colorspace. Each spectrum is converted into color coordinates using the, D65 illumination for the calculations, and CIE 1931 2 Degree Standard Observer color-matching functions. A $\Delta E_{00}$ of around 2 is a just noticeable color difference for a human observer.

The second spectral reconstruction metric is the root mean square error (RMSE) between the reference spectrum and a reconstructed spectrum. There is no need to include the goodness of fit coefficient (GFC) or the angular error, since previous studies [54] have shown that these correlate strongly with the RMSE.

### 3.5. Application-Specific Metric and Oxygenation Level Estimation

The third metric is a validation of the oxygenation level estimations. This parameter can be approximated through calculations using the reflectance spectrum of skin. The reflectance spectrum of skin is the result of concentrations of particular chromophores present in the skin. The ratio between oxygenated and deoxygenated hemoglobin reflects the relative oxygenation level in the skin and is an important parameter for diagnostics. Hemoglobin occurs in different forms but only these two are relevant for oxygenation. Different methods have been proposed to estimate oxygenation levels from particular wavelengths [27,29,49,55].

For this study, the estimation uses a multiple regression method described by Nishidate et al. [49]. A fast way of estimating absorbance A(λ) from reflectance assumes the Lambert-Beer law:

(5) $$A\left(\lambda \right)=-log_{10}R\left(\lambda \right)$$

According to the simplified Lambert-Beer law the total absorbance of skin tissue can be described with:

(6) $$\begin{array}{c} \begin{array}{c} A\left(\lambda \right)=C_{m}l_{e}\left(\lambda ,C_{m}\right)\varepsilon_{m}\left(\lambda \right)+C_{bi}l_{d}\left(\lambda ,C_{bi}\right)\varepsilon_{bi}\left(\lambda \right)+C_{ob}l_{d}\left(\lambda ,C_{ob},C_{db}\right)\varepsilon_{ob}\left(\lambda \right)+\\ C_{db}l_{d}\left(\lambda ,C_{ob},C_{db}\right)\varepsilon_{db}\left(\lambda \right), \end{array} \end{array}$$

where $\varepsilon_{m},\varepsilon_{b},\varepsilon_{ob}$, $\varepsilon_{db}$ describe the molar extinction coefficients of melanin, bilirubin, oxygenated and deoxygenated hemoglobin and $C_{m},C_{b},C_{ob}$, $C_{db}$ describe the concentration of each specific chromophore. $l_{e}$ describes the mean optical path length for epidermis, $l_{d}$ for dermis and D(λ) describes the attenuation due to scattering these values are taken from literature. This equation can be solved by multiple regression analysis and is therefore reformulated to:

(7) $$\begin{array}{c} \begin{array}{cc} A(\lambda_{1}) & =c_{m}\varepsilon_{m}\left(\lambda_{1}\right)+c_{bi}\varepsilon_{bi}\left(\lambda_{1}\right)+c_{ob}\varepsilon_{ob}\left(\lambda_{1}\right)+c_{db}\varepsilon_{db}\left(\lambda_{1}\right)\\ A(\lambda_{2}) & =c_{m}\varepsilon_{m}\left(\lambda_{2}\right)+c_{bi}\varepsilon_{bi}\left(\lambda_{2}\right)+c_{ob}\varepsilon_{ob}\left(\lambda_{2}\right)+c_{db}\varepsilon_{db}\left(\lambda_{2}\right)\\ A(\lambda_{3}) & =c_{m}\varepsilon_{m}\left(\lambda_{3}\right)+c_{bi}\varepsilon_{bi}\left(\lambda_{3}\right)+c_{ob}\varepsilon_{ob}\left(\lambda_{3}\right)+c_{db}\varepsilon_{db}\left(\lambda_{3}\right)\\ .\\ .\\ A(\lambda_{n}) & =c_{m}\varepsilon_{m}\left(\lambda_{n}\right)+c_{bi}\varepsilon_{bi}\left(\lambda_{n}\right)+c_{ob}\varepsilon_{ob}\left(\lambda_{n}\right)+c_{db}\varepsilon_{db}\left(\lambda_{n}\right), \end{array} \end{array}$$

where $c_{m},c_{b}i,c_{ob},c_{db}$ are closely related to the concentrations of melanin, bilirubin, oxygenated and deoxygenated blood and represent the unit-less contribution of each extinction coefficient to the total absorbance *A*. Any number of wavelengths can be used to calculate the absorbances. Reflectance spectra can be converted to absorbance spectra according to Equation (5) and then used with the following equation. The calculation of the concentration of any chromophore can then be formulated in matrix notation as:

(8) $$\begin{array}{c} \begin{array}{cc} a & =\varepsilon c\\ c & =\varepsilon^{-1}a \end{array} \end{array}$$

Finally, oxygen saturation can be calculated with:

(9) $$S_{oxy}=\frac{C_{ob}}{C_{ob}+C_{db}}$$

Even though a simplification of the physical light skin interactions, methods based on these principles have been used for oxygenation level estimation [49,56,57,58]. This approach allows rapid calculation of tissue parameters with low computational complexity. It is assumed that most other chromophores are constant over time. The oxygenation of blood is not constant, due to oxygen consumption by tissue. According to Equation (9) oxygenation level estimation is calculated using both the reflectance spectra and reconstructed spectra. The Euclidean distance between the two resulting oxygenation level estimation values is then calculated and used as a quality metric to judge the reconstruction accuracy with:

(10) $$\Delta Oxy=|S_{oxy1}-S_{oxy2}|$$

## 4. Experimental Setup

This section will be discussing the concrete choices of sensors, training and test data, and finally, summarize the approach. A new database of simulated skin spectra is also created and described in detail in this section.

### 4.1. Sensors

Five cameras are investigated the Sinarback 54 RGB camera (RGB) as representative for common three-channel imaging, three spectral filter array cameras are considered, XIMEA xiSpec MQ022HG-IM-SM4X4-VIS [4,5], Silios technologies CMC-C [6] (Silios) and a prototypical device by Thomas et al. [3] (France1). Table 1 provides an overview of their key features and is sorted by the number of bands. The CorXim ’virtual cameras is added, which is the corrected version of the Ximea xispec [4] camera by applying a linear transformation matrix provided by the manufacturer which reduces the effect of secondary transmission peaks in some filter bands [59]. It is considered to be an independent camera to test the impact of such a correction.

**Figure 3:**
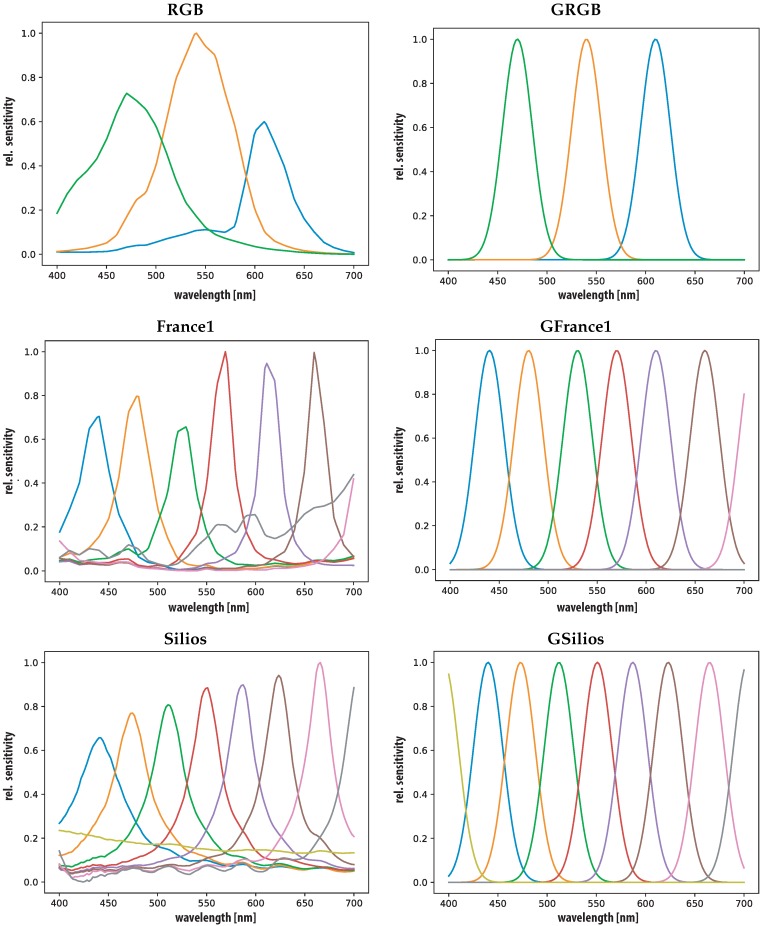
Sensor sensitivities, one RGB camera [60], a prototypical implementation by Thomas et al. [3] (*France1*) and commercially available Silios [6] (Silios) (all **left**) and simulated GSB (GRGB, GFrance1 and GSilios) versions (all **right**).

**Figure 4:**
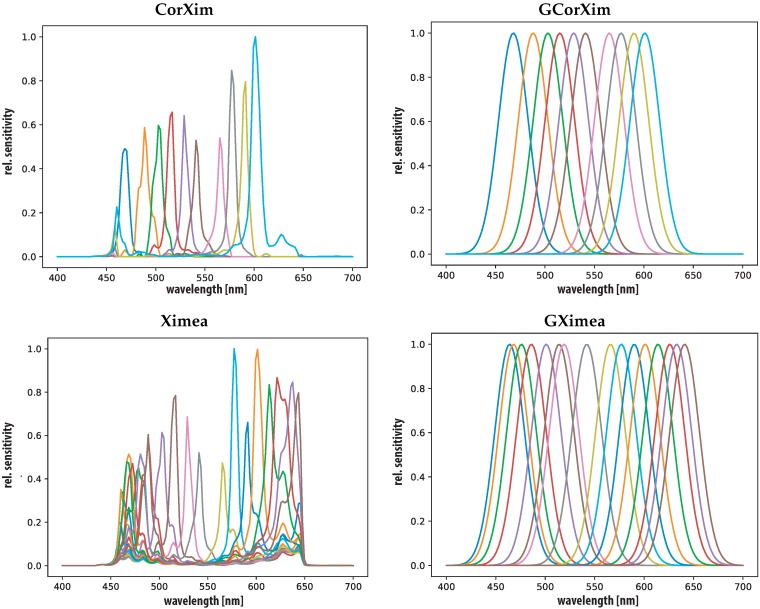
Sensor sensitivities, Ximea xispec [4] (Ximea and CorXim) (all **left**) and simulated GSB (GCorXim and GXimea) versions (all **right**).

Figure 3 and Figure 4 show the spectral sensitivities of all cameras in the spectral range of (400–700 nm) with a measurement interval of 2 nm steps. All sensitivities are measured and provided by the camera manufacturers and interpolated to this range and measurement interval. Additionally, for each camera a virtual GSB sensor is generated and included in the study. The GSB are generated according to Thomas [52] at each of the sensitivity peaks of each camera(GRGB, GFrance1, GSilios, GCorXim, GXimea). All GSB have a σ = 15 nm and provide a virtual version of each camera with perfectly shaped narrow band sensitivities.

### 4.2. Generating a Training Set

The skin simulations are generated using a modification of the multi-layered Monte Carlo tissue model (MCML) published by Atencio et al. [39]. This code was modified to vary and simulate combinations chromophore concentrations and blood volume fractions [61]. Changing Bilirubin concentration $C_{bi}$, oxygen saturation $S_{oxy}$, blood and melanin volume fractions $f_{bl}$ and $f_{mel}$ were changed and 10,000 skin reflectances were calculated.

This simulation environment is based on a three-layer skin model and initially proposed to simulate bilirubin concentration in the skin of the forehead of newborns. The three layers are epidermis, dermis and a bone layer. This model assumes each of the layers as infinite homogenous media with a defined absorption per layer. Scattering is assigned uniformly to both layers. Each layer has different chromophores contributing to its absorption based on the volume fractions of melanin ($f_{mel})$ blood, ($f_{bl}$) and bilirubin ($f_{bi}$). Epidermis contains melanin, dermis contains bilirubin and oxy- deoxygenated hemoglobin. The total absorbance of each of the layers is the sum of the absorbance fractions of chromophores present in that particular layer and defined as $\mu_{a}$. The chromophore parameters for the Monte Carlo simulation, were chosen to cover the entire range defined by Atencio et al. [39] (see Table 2). For melanin volume fractions of approximately 1% to 6.3% equivalent to fair skin according to Jacques [62].

Each of the chromophore absorption coefficients is modelled from the data provided by Jacques et al. [63]. This $\mu_{a}$ can be seen as analogous to the Absorption *A* in previous equations, but in the context of defining optical properties of skin it is referred to as $\mu_{a}$. For the epidermis, the absorption $\mu_{a_{e}}$ only depends on melanin the only chromophore present in this layer with:

(11) $$\mu_{a_{epi}}=f_{mel}\mu_{a_{mel}}\left(\lambda \right)$$

The Monte Carlo simulation framework by Atencio et al. mentions specifically that the model needs further testing and verification to simulate darker skin types, therefore even higher melanin volume fractions were not included as parameters for the simulation. To calculate the final absorption of the dermis layer both bilirubin and blood are the main contributors:

(12) $$\mu_{a_{derm}}=f_{bl}\mu_{a_{bl}}\left(\lambda \right)+f_{bi}\mu_{a_{bi}}\left(\lambda \right)$$

$f_{bi}$ is considered to be constant and the parameter is the concentration of bilirubin as:

(13) $$\mu_{a_{bil}}\left(\lambda \right)=ln\left(10\right)\frac{C_{bi}}{P_{Mbi}}\epsilon_{bi}\left(\lambda \right),$$

where $P_{Mbi}$ is a constant and $\epsilon_{bi}\left(\lambda \right)$ are the literature values for the extinction coefficients for bilirubin [63]. $f_{bl}$ describes the volume fraction of total blood in the dermis layer. The volume fraction parameters for this simulation cover typical values homogeneously distributed blood in the dermis layer [63]. Due to differences in absorbance for oxygenated hemoglobin and deoxygenated hemoglobin $\mu_{a_{blo}}\left(\lambda \right)$ is calculated as:

(14) $$\mu_{a_{blo}}=S\mu_{a_{ob}}\left(\lambda \right)+\left(1-S\right)\mu_{a_{db}}\left(\lambda \right)$$

*S* describes the oxygen saturation in the blood and is to be estimated. The dataset will be verified in the Results Section 5 using a principle component analysis.

## 5. Results and Discussion

### 5.1. Training Set Validation

The first results presented in this study address the skin simulation database and can be seen as an additional verification for using this simulated training set. It is based on principle component analysis (PCA) of the sets included in this research.

The principle components allow representation of the multidimensional set in a lower-dimensional space. If the principle components are calculated for a combined set they represent the orthogonal axes of a space describing the sets. The area covered by the sets plot into this orthogonal space describes the diversity of the particular set. If multiple sets are plot into the same principle component space the difference in diversity and area covered within that PCA space can be analyzed.

**Figure 5:**
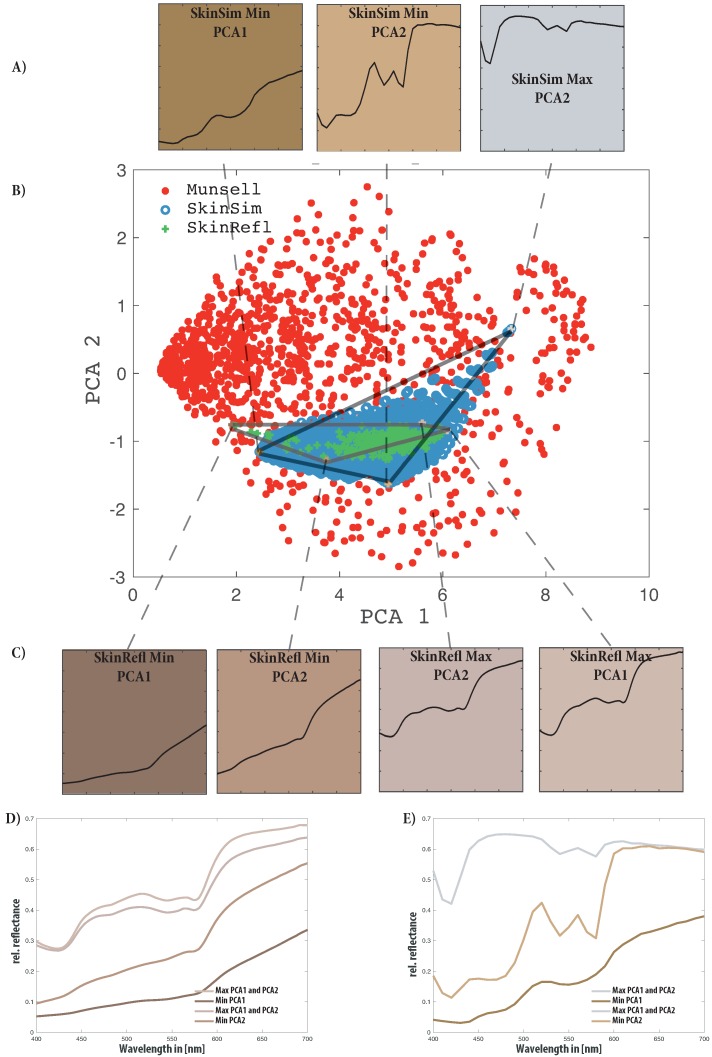
Dimensionality analysis of all sets combined (**B**) skin simulation (blue), skin reflectance (green) and Munsell reflectances (red). Colored markings for maximum PCA1, minimum PCA1, maximum PCA2, minimum PCA2, for skin simulation and skin reflectance, respectively. Color patch recreation (under D65 light source) of the extreme spectra for the skin simulation (**A**) and skin reflectance database (**C**) with minimum PCA 1 and PCA 2 and maximum PCA 1 and PCA 2. Plot of the maximum and minimum spectra for the skin reflectance database (**D**) and skin simulation database (**E**) according to PCA analysis.

The sets are shown along the first two principal components of the combined set in Figure 5. Table 3 shows the resulting principle components of each of the sets and the combined set. The Munsell set is the most diverse considering its low first principle component. The skin simulation set covers a wider range of reflectances compared to measured skin reflectances. This is represented in a lower first principle component. Physiological parameters cover a wider range than living tissue see Table 2.

In Figure 5 it can be observed that the skin simulation covers all the measured skin reflectances except for a few measurements. This can be ascribed to the limited number of parameters for the simulation, resulting in some measured skin reflectances not being represented within the skin simulation. The skin model is limited to Caucasian fair skin and initially designed for neonatal babies. To further analyze the parameter of the skin simulation, which falls far out of the measured skin reflectance, the extreme curves where plotted.

Figure 5D,E shows these extreme curves of both the skin reflectance and the skin simulation set as marked in Figure 5A. In Table 4 it becomes apparent that the main factor for the simulations is the blood volume parameter. All extreme results according to the PCA analysis have an extreme value for the blood volume. The melanin parameter also contributes to extreme values within the principle component space indicating the strong influence of melanin on the resulting skin spectra. In this principle component space, the bilirubin concentration parameter spreads the distribution of points.

Figure 5 also contains sRGB [64] color swatches reproduced under a virtual D65 illumination. These provide a visual impression of the color of the extreme points in the principle component space. They show that the extreme value curves, not included within the skin simulation represent darker skin types and that extreme values of the skin simulation can include physiologically unlikely scenarios of grey skin.

### 5.2. Spectral Reconstruction

**Figure 6:**
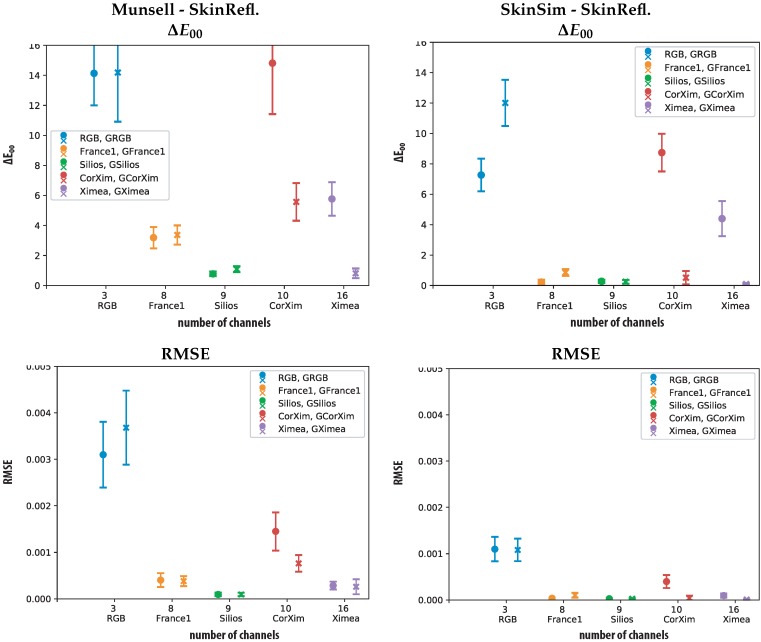
Resulting metrics of $\Delta E_{00}$ (D65, CIE 2${}^{\circ }$ 1931) (**top**) and RMSE calculated between reconstruction and training (**bottom**). All Sensors, Munsell set (**left**) and Skin Simulation set (**right**) as training including standard deviation of the resulting data. For all graphs, the filled “o” represents the original sensor and the “x” represents the GSB.

Results for the two spectral reconstruction metrics calculated for each of the four sensors and their simulated GSB versions are shown in Figure 6. Each of the graphs shows mean results and standard deviation of the actual sensor as a circle and the GSB sensor results as a cross. All metrics are calculated with the different training sets (Munsell and skin simulation) for the spectral reconstruction and plotted. The cartesian coordinate system consists of the number of channels on the x-axes and the value for each of the metrics on the y-axes.

These plots allow the comparison between the sensors according to the different metrics in two scenarios. It can be observed that the performance in RMSE and $\Delta E_{00}$ correspond to each other.

Figure 6 provides a plot of the $\Delta E_{00}$ difference between the test reflectances and their reconstruction. Surprisingly, the plots show that the corrected Ximea performs the worst in the case of Munsell patches for training and according to $\Delta E_{00}$. This can be ascribed to the cut of spectral sensitivity imposed by the linear correction transformation. Figure 4 shows the low sensitivity of this sensor at the edges of the chosen spectral range (400 nm to 700 nm).

**Figure 7:**
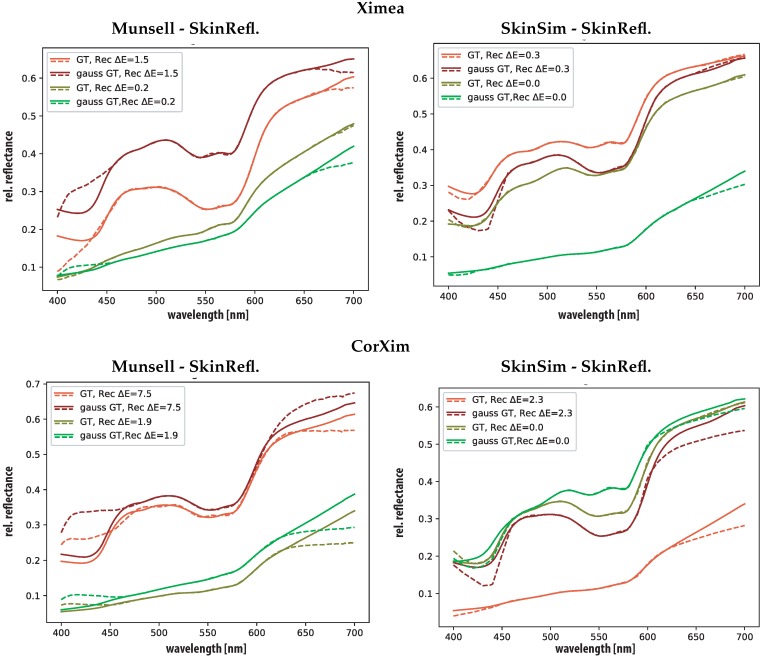
Visualization of worst and best $\Delta E_{00}$ results for the uncorrected Ximea (Ximea top) and corrected Ximea (CorXim bottom), Munsell set (**left**) and Skin simulation (SkinSim) set (**right**) as training. Each graph includes GSB sensor results, ground-truth in solid lines and estimation with dashed lines.

Figure 7 contains plots of the spectral reflectances ground-truth and reconstructed that are responsible for the highest $\Delta E_{00}$ results for the corrected and uncorrected Ximea camera. The plot allows appreciation of the areas of the spectra that cause high $\Delta E_{00}$ results. In the case of the corrected Ximea camera spectral regions that have low or zero sensitivity are wrongly reconstructed. This is not surprising but confirms the poorer performance of the corrected Ximea camera in comparison with the uncorrected Ximea camera in the $\Delta E_{00}$ and RMSE metric. The more limited spectral coverage of the corrected spectral imager negatively influences the spectral reconstruction ability of this camera.

The second worst performer regarding color differences (Mean $\Delta E_{00}$ = ~14 and Mean $\Delta E_{00}$ = ~12) is the RGB camera. Both the low number of channels and their specific overlap in the spectral region seems to influence the estimation accuracy negatively. The lower performance of the GSB version can be ascribed to the low sigma (σ=15) of the gaussian filters. In the case of the RGB sensor, the coverage of the spectral range of interested is as seen in Figure 3 not optimal. The spectral distribution shows significant areas of very low spectral sensitivity and negatively influences the spectral reconstructions.

Both corrected (CorXim) and uncorrected Ximea benefit greatly from GSB improving the performance according to the $\Delta E_{00}$ metric. For the Silios camera, the GSB only improve the $\Delta E_{00}$ performance when using the expert training set as the skin simulation set. One explanation could be the sharp cut off for the GSB resulting from the bands that exceed the spectral range of this analysis. The prototypical sensor *France1* has initially already close to gaussian sensitivities and does not benefit from the GSB.

The RMSE metric shows a similar trend compared to $\Delta E_{00}$. The Ximea camera scores better results regarding the RMSE in comparison with $\Delta E_{00}$. Differences between original sensors and GSB sensors are smaller considering this metric.

Training theWiener estimation matrix with the proposed specialized skin simulation set results in a more robust reconstruction according to $\Delta E_{00}$ and RMSE for all tested cameras. The more general Munsell set lacks skin spectral shapes and is contains two dissimilar spectra in comparison with the skin test set. The similar shapes and increased number of spectra in the generated specialized database improve the spectral reconstructions.

### 5.3. Oxygenation Level Estimation

**Figure 8:**
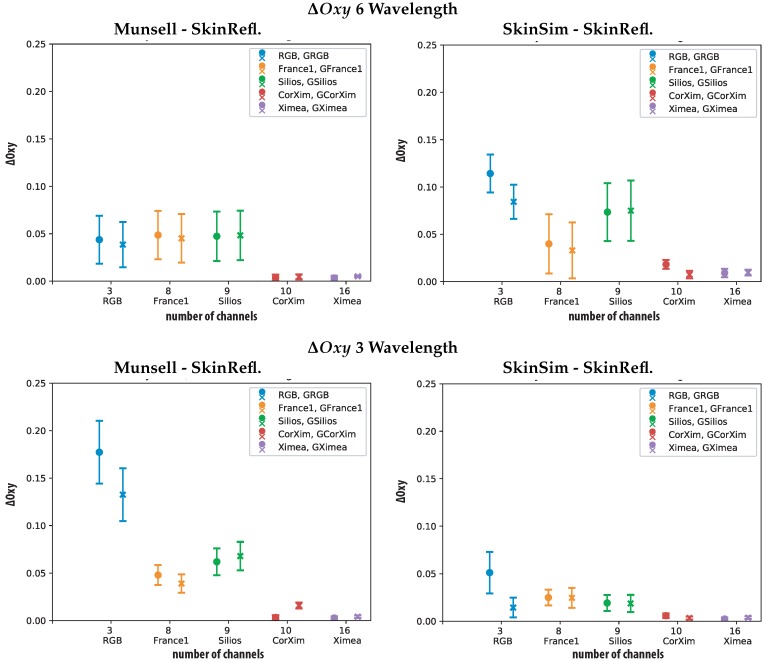
Resulting values for ΔOxy metric calculated using six wavelength (500 nm, 520 nm, 540 nm, 560 nm, 580 nm, 600 nm) (**top**) and three wavelengths (480 nm, 560 nm, 600 nm) (**bottom**) for all Sensors. Munsell set (**left**) as training and Skin Simulation set (**right**) including standard deviation of the data.

The oxygenation estimations were performed using six wavelengths as proposed by Nishidate et al. (500 nm, 520 nm, 540 nm, 560 nm, 580 nm, 600 nm) and three wavelengths (480 nm, 560 nm, 600 nm) the results are shown in Figure 8.

These two oxygenation metrics show different behavior for all cameras compared to the spectral accuracy metrics. The eight and nine channel cameras (*France1* and Silios) perform the worst for the Munsell training case and six wavelengths. This is surprising since these two cameras perform the best according to the spectral reconstruction metrics $\Delta E_{00}$ and RMSE. For this case, the performance differences between the GSB sensor and the original sensor are very small. One explanation can be that these key wavelengths all fall into valleys between the sensitivity peaks for the Silios and *France1* sensor. The GSB sensors could be affected equally or stronger, due to the relatively small sigma (σ=15).

The wavelengths proposed by Nishidate et al. are optimized for an RGB sensor. For the specialized training set, the RGB camera performs the worst. Illustrating that the spectral reconstruction using a specialized training set benefits from narrow spectral channels.

Figure 8 also contains results for the oxygenation metric using three wavelengths (480 nm, 560 nm, 600 nm). It can be observed that the choice of the training set for this configuration influences the different cameras independently. For Munsell patch training, the RGB camera performs the worst and both versions of the Ximea camera the best. Using the specialized training set the differences between all cameras are smaller and the RGB camera still performs worst. The other sensors are less affected by the change of training sets only slightly lowering their oxygenation metric differences when using the specialized training set. For the idealized GSB RGB sensor lower oxygenation metric differences can be observed compared to some of the SFA sensors. This could be ascribed to the wavelength chosen for oxygenation level estimation which all fall well within high sensitivity of the gaussian RGB (GRGB) sensor.

A camera with sensitivity peaks at the wavelength of interest should perform optimally. This can be used if the wavelength of interest are known. None of the investigated cameras has optimal filter sensitivity peaks for oxygenation estimation. Table 5 provides an overview of the statistical results for all sensors, considering the better performing skin simulation training data set.

The proposed specialized training set improved the final oxygenation parameters (estimated with three wavelengths). In the case of six wavelengths the skin training set performs worse than the Munsell set. One explanation is that using six wavelengths includes wavelengths at the outer edges of the considered spectral range. The specialized set provides too little variety for these areas and the diverse Munsell set trains these regions better.

For future work noise should be incorporated into the framework. The chosen wiener estimation method has room to incorporate a noise term into the spectral estimation and the impact of different kind of noise should be studied. The framework also allows simulation and comparison of spectral filter array cameras in different spectral ranges. Near infrared should be considered for future work as it is used in traditional oximetry systems. Furthermore, oxygenation estimation methods that use the full spectra based on inverse Monte Carlo methods should be tested in conjunction with spectral reflectance reconstruction.

### 5.4. Summary and Conclusions

A straightforward framework to evaluate spectral filter array cameras based on spectral sensitivities and publicly available skin and reflectance databases was proposed. It allows to compare and quantify the performance of SFA cameras for medical applications and skin imaging in particular. The framework does not require prior measurements and is based on a readily available skin databases for testing, a proposed generated skin simulation database and sensor sensitivities of the cameras included.

Reconstructing full reflectances from sensor responses allows to comparison and is useful when the application-specific bands of interest are unknown. It can be useful to recreate color images and benefits from a specialized training set. If the bands of interest are known a camera with high sensitivity for those exact bands is advisable. Several observations particular to spectral filter array cameras were made:

- Spectral shapes of the filters should be adapted application-specific
- Careful choice of the spectral bands should be adapted application-specific
- Selecting an optimal training set for spectral reflectances reconstruction improves the results for SFAs with narrow spectral sensitivities
- GSB improve spectral reconstruction considering $\Delta E_{00}$ color differences and RMSE
- GSB have a small impact on oxygenation level estimation if the bands are not close to the ideal wavelength for oxygen estimation

The framework has been applied to compare commercially available SFA cameras for skin diagnosis and skin oxygenation level estimation.

The corrected Ximea camera performed the best in terms of oxygenation level estimations. Regarding the spectral reconstruction and $\Delta E_{00}$ color difference metrics the Silios camera shows the best results. Recommending it for applications where the specific bands of interest are not known.

SFA cameras hold great potential for monitoring vital functions and medical diagnosis as a non-contact, real-time spectral imaging modality. This framework provides a basis for using spectral filter array cameras effectively for medical applications. It can be used to design spectral filter sensitivities for specific applications by optimizing the wavelength bands and transmission shapes of the filters. It is, however, necessary to verify the findings with experimental data and extend the framework to include spatial aspects.

## Figures and Tables

**Table 1:** Features of the included cameras. RGB camera [60], commercially available XIMEA Xispec SFA camera [4,5] (Ximea and CorXim), Silios technologies SFA camera [6] (Silios), and a prototypical device from academia [3] (France1).

Property	RGB	France1	Silios	CorXim	Ximea
spectral bands	3	8	9	10	16
spectral peak range [nm]	480–610	440–850	445–710	465–630	465–630
frame rate [Hz]	60	60	60	170	170
resolution per band	4080×5440	160×128	426×339	512×272	512×272
size [mm]	38.8×50.0	NA	56×56×22	26×26×26	26×26×26

**Table 2:** Parameter range for MCML (Monte Carlo modelling of light transport in multi-layered tissues) skin simulation [39,61] resulting in 10,000 different parameter combinations. $S_{oxy}$ is the saturation of oxygenation, $f_{bl}$ and $f_{mel}$ the volume fraction of blood and bilirubin, and $C_{bi}$ describes the bilirubin concentration. Green and red Shadings indicate extreme values of simulation range.

Parameter	Level: 1	2	3	4	5	6	7	8	9	10
$S_{oxy}$	10%	20%	30%	40%	50%	60%	70%	80%	90%	100%
$f_{bl}$	0.1%	0.2%	0.3%	0.4%	0.5%	0.6%	0.7%	0.8%	0.9%	1%
$C_{bi}$	0.0	0.025	0.05	0.075	0.1	0.125	0.15	0.175	0.2	0.225
$f_{mel}$	0	2%	3%	4 %	5%	6%	7%	8%	9%	10%

**Table 3:** Resulting PCAs for all sets. Variance of each of the sets along the first 4 principle components.

PCA	Munsell	SkinSim	SkinRefl	Combined
1	76.8	87.1	96.0	74.7
2	15.8	7.7	2.1	17.0
3	6.0	4.3	1.5	4.8
4	0.8	0.5	0.2	2.1

**Table 4:** Monte Carlo Simulation parameters for the extreme points according to the principle components. Red background items indicate the maximum of their particular parameter, while green background indicate minima for the range of input parameters.

Parameter	Max PCA1 and PCA2	Min PCA1	Min PCA2
$StO_{2}Saturation$	10%	100 %	10%
$f_{Blood}$	0.1%	1%	1%
$C_{Bilirubin}$	0.225	0.0	0.225
$f_{Melanin}$	0.0 %	10%	10%

**Table 5:** Statistical results (minimum, maximum, mean, standard deviation, 98%) for all sensors for $\Delta E_{00}$ (top), RMSE (2nd from top), ΔOxy 6wvl (3rd from top) and ΔOxy 3wvl (bottom). All values are based on skin simulation set as training and skin reflectance set as testing.

$\Delta E_{00}$												
Sensor	Min	Max	Mean	Std	98%			Min	Max	Mean	Std	98%
RGB	5.04	11.03	7.27	1.08	9.20		GRGB	8.89	16.76	12.01	1.52	14.87
*France1*	0.02	0.93	0.22	0.15	0.68		*GFrance1*	0.40	1.50	0.86	0.23	1.35
Silios	0.04	0.66	0.28	0.11	0.49		GSilios	0.03	0.75	0.25	0.12	0.51
CorXim	5.82	11.99	8.74	1.24	11.50		GCorXim	0.02	2.27	0.51	0.44	2.18
Ximea	0.89	6.81	4.40	1.16	6.38		GXimea	0.00	0.30	0.09	0.07	0.25
**RMSE**												
**Sensor**	**Min**	**Max**	**Mean**	**Std**	**98%**			**Min**	**Max**	**Mean**	**Std**	**98%**
RGB	0.000647	0.002112	0.001099	0.000263	0.001717		GRGB	0.00067	0.00194	0.00108	0.00024	0.00180
*France1*	0.00001	0.00009	0.00004	0.00001	0.00007		*GFrance1*	0.00003	0.00037	0.00010	0.00005	0.00022
Silios	0.000003	0.00006	0.00003	0.00001	0.00005		GSilios	0.000003	0.00006	0.00003	0.00001	0.00006
CorrXim	0.000184	0.00099	0.00040	0.00014	0.00081		GCorXim	0.000004	0.00028	0.00004	0.00005	0.00026
Ximea	0.000007	0.00028	0.00010	0.00004	0.00020		GXimea	0.000002	0.00003	0.00001	0.00001	0.00003
**Oxyg. Metric 6wvl**												
**Sensor**	**Min**	**Max**	**Mean**	**Std**	**98%**			**Min**	**Max**	**Mean**	**Std**	**98%**
RGB	0.070	0.169	0.114	0.020	0.155		GRGB	0.051	0.125	0.084	0.018	0.119
*France1*	0.001	0.150	0.040	0.031	0.109		*GFrance1*	0.0001	0.145	0.033	0.030	0.102
Silios	0.002	0.140	0.073	0.031	0.131		GSilios	0.006	0.151	0.075	0.032	0.134
CorXim	0.001	0.028	0.018	0.005	0.027		GCorXim	0.0002	0.017	0.007	0.004	0.016
Ximea	0.000	0.019	0.009	0.004	0.018		GXimea	00.002	0.017	0.009	0.003	0.016
**Oxyg. Metric 3wvl**												
**Sensor**	**Min**	**Max**	**Mean**	**Std**	**98%**			**Min**	**Max**	**Mean**	**Std**	**98%**
RGB	0.010	0.132	0.051	0.022	0.090		GRGB	0.0001	0.051	0.014	0.010	0.044
*France1*	0.001	0.041	0.025	0.008	0.038		*GFrance1*	0.002	0.048	0.025	0.010	0.043
Silios	0.001	0.043	0.019	0.008	0.035		GSilios	0.001	0.048	0.019	0.009	0.036
CorXim	0.00004	0.011	0.006	0.002	0.010		GCorXim	0.0001	0.008	0.003	0.002	0.007
Ximea	0.001	0.041	0.025	0.008	0.038		GXimea	0.00001	0.007	0.004	0.001	0.006

## Abbreviations

The following abbreviations are used in this manuscript:

SFA	spectral filter array
GFC	goodness of fit coefficient
RMSE	root mean square error
sRGB	standard RGB
MCML	Monte Carlo modelling of light transport in multi-layered tissues
GSB	gaussian spectral bands

## References

[1] Lapray P.J., Wang X., Thomas J.B., Gouton P. (2014). Multispectral filter arrays: Recent advances and practical implementation. Sensors.

[2] Ewerlöf M., Larsson M., Salerud E.G. (2017). Spatial and temporal skin blood volume and saturation estimation using a multispectral snapshot imaging camera. Proc. SPIE.

[3] Thomas J.B., Lapray P.J., Gouton P., Clerc C. (2016). Spectral Characterization of a Prototype SFA Camera for Joint Visible and NIR Acquisition. Sensors.

[4] Ximea (2018). Hyperspectral Cameras. https://www.ximea.com.

[5] IMEC (2018). Hyperspectral-Imaging. https://www.imec-int.com.

[6] SILIOS (2018). Multispectral-Imaging. https://www.silios.com.

[7] Pedersen M., Hardeberg J.Y. (2012). Full-reference image quality metrics: Classification and evaluation. Found. Trends® Comput. Graph. Vis..

[8] Wang Z., Bovik A.C., Sheikh H.R., Simoncelli E.P. (2004). Image Quality Assessment: From Error Visibility to Structural Similarity. IEEE Trans. Image Process..

[9] Chandler D.M. (2013). Seven Challenges in Image Quality Assessment: Past, Present, and Future Research. ISRN Signal Process..

[10] Miao L., Qi H., Ramanath R., Snyder W.E. (2006). Binary tree-based generic demosaicking algorithm for multispectral filter arrays. IEEE Trans. Image Process..

[11] Monno Y., Tanaka M., Okutomi M. Multispectral demosaicking using adaptive kernel upsampling. Proceedings of the 2011 18th IEEE International Conference on Image Processing.

[12] Wang C., Wang X., Hardeberg J.Y. (2014). A Linear Interpolation Algorithm for Spectral Filter Array Demosaicking. Image and Signal Processing.

[13] Wang X., Thomas J.B., Hardeberg J.Y., Gouton P. A Study on the Impact of Spectral Characteristics of Filters on Multispectral Image Acquisition. Proceedings of the 12th Congress of the International Colour Association.

[14] Park C., Kang M. (2016). Color Restoration of RGBN Multispectral Filter Array Sensor Images Based on Spectral Decomposition. Sensors.

[15] Nazari R.M. (2017). Denoising and Demosaicking of Color Images. Ph.D. Thesis.

[16] Bersha K.S. (2010). Spectral Imaging and Analysis of Human Skin. Master’s Thesis.

[17] Kuzmina I., Diebele I., Jakovels D., Spigulis J., Valeine L., Kapostinsh J., Berzina A. (2011). Towards noncontact skin melanoma selection by multispectral imaging analysis. J. Biomed. Opt..

[18] Nishidate I., Tanaka N., Kawase T., Maeda T., Yuasa T., Aizu Y., Yuasa T., Niizeki K. (2011). Noninvasive imaging of human skin hemodynamics using a digital red-green-blue camera. J. Biomed. Opt..

[19] Jakovels D., Spigulis J. (2012). RGB imaging device for mapping and monitoring of hemoglobin distribution in skin. Lith. J. Phys..

[20] Jakovels D., Kuzmina I., Berzina A., Spigulis J. (2012). RGB imaging system for monitoring of skin vascular malformation’s laser therapy. Proc. SPIE.

[21] Kumar A., Dhawan A.P., Relue P., Chaudhuri P.K. Multi-spectral optical imaging of skin to diagnose malignant melanoma. Proceedings of the Engineering in Medicine and Biology.

[22] Cotton S., Claridge E., Hall P. A skin imaging method based on a colour formation model and its application to the diagnosis of pigmented skin lesions. Proceedings of the Medical Image Understanding and Analysis.

[23] Tsumura N., Kawabuchi M., Haneishi H., Miyake Y. (2000). Mapping Pigmentation in Human Skin by Multi-Visible-Spectral Imaging by Inverse Optical Scattering Technique. Color Imaging Conf..

[24] Balas C., Themelis G., Papadakis A., Vasgiouraki E. A novel hyper-spectral imaging system: Application on in-vivo detection and grading of cervical precancers and of pigmented skin lesions. Proceedings of the IEEE Computer Society Workshop on Computer Vision Beyond the Visible Spectrum.

[25] Kerekes J., Subramanian N., Kearney K., Schad N. (2006). Spectral imaging of skin: Experimental observations and analyses. Proc. SPIE.

[26] Randeberg L.L., Baarstad I., Løke T., Kaspersen P., Svaasand L.O. (2006). Hyperspectral imaging of bruised skin. Proc. SPIE.

[27] Klaessens J.H.G.M., Noordmans H.J., de Roode R., Verdaasdonk R.M. (2009). Non-invasive skin oxygenation imaging using a multi-spectral camera system: Effectiveness of various concentration algorithms applied on human skin. Proc. SPIE.

[28] Spigulis J., Jakovels D., Rubins U. (2010). Multi-spectral skin imaging by a consumer photo-camera. Proc. SPIE.

[29] Huang J. (2013). Multispectral Imaging of Skin Oxygenation. Ph.D. Thesis.

[30] Poxon I., Wilkinson J., Herrick A., Dickinson M., Murray A. (2014). Pilot study to visualise and measure skin tissue oxygenation, erythema, total haemoglobin and melanin content using index maps in healthy controls. Proc. SPIE.

[31] Van Gastel M., Stuijk S., De Haan G. (2016). New principle for measuring arterial blood oxygenation, enabling motion-robust remote monitoring. Sci. Rep..

[32] Bauer J.R., van Beekum K., Klaessens J.H.G.M., Noordmans H.J., Boer C., Hardeberg J.Y., Verdaasdonk R.M. (2018). Towards real-time non contact spatial resolved oxygenation monitoring using a multi spectral filter array camera in various light conditions. Proc. SPIE.

[33] Preece S.J., Claridge E. (2004). Spectral filter optimization for the recovery of parameters which describe human skin. IEEE Trans. Pattern Anal. Mach. Intell..

[34] Gutiérrez-Gutiérrez J., Pardo A., Real E., López-Higuera J., Conde O.M. (2019). Custom Scanning Hyperspectral Imaging System for Biomedical Applications: Modeling, Benchmarking, and Specifications. Sensors.

[35] Saager R.B., Baldado M.L., Rowland R.A., Kelly K.M., Durkin A.J. (2018). Method using in vivo quantitative spectroscopy to guide design and optimization of low-cost, compact clinical imaging devices: Emulation and evaluation of multispectral imaging systems. J. Biomed. Opt..

[36] Jimenez J., Scully T., Barbosa N., Donner C., Alvarez X., Vieira T., Matts P., Orvalho V., Gutierrez D., Weyrich T. (2010). A practical appearance model for dynamic facial color. ACM Trans. Graph. (Proc. SIGGRAPH Asia).

[37] Iglesias-Guitian J.A., Aliaga C., Jarabo A., Gutierrez D. (2015). A Biophysically-Based Model of the Optical Properties of Skin Aging. Comput. Graph. Forum.

[38] Lapray P.J., Thomas J.B., Gouton P. (2017). High Dynamic Range Spectral Imaging Pipeline For Multispectral Filter Array Cameras. Sensors.

[39] Delgado Atencio J.A., Jacques S.L., Montiel S.V. (2011). Monte Carlo Modeling of Light Propagation in Neonatal Skin.

[40] Hiltunen J. (2019). Munsell Book of Color: Matte Finish Collection Measured by J. Hiltunen. https://www.uef.fi/web/spectral/munsell-colors-matt-spectrofotometer-measured.

[41] Munsell Color (1976). Munsell Book of Color: Matte Finish Collection.

[42] Cooksey C.C., Allen D.W., Tsai B.K. (2017). Reference Data Set of Human Skin Reflectance. J. Res. Natl. Inst. Stand. Technol..

[43] Publication, CIE (2004). CIE 15: Technical Report: Colorimetry.

[44] Hardeberg J.Y. (1999). Acquisition and Reproduction of Color Images: Colorimetric and Multispectral Approaches. Ph.D. Thesis.

[45] Imai F.H., Berns R.S. Spectral estimation using trichromatic digital cameras. Proceedings of the International Symposium on Multispectral Imaging and Color Reproduction for Digital Archives.

[46] Shimano N., Terai K., Hironaga M. (2007). Recovery of spectral reflectances of objects being imaged by multispectral cameras. J. Opt. Soc. Am. A.

[47] Shimano N., Hironaga M. (2010). Recovery of spectral reflectances of imaged objects by the use of features of spectral reflectances. J. Opt. Soc. Am. A.

[48] Stigell P., Miyata K., Hauta-Kasari M. (2007). Wiener estimation method in estimating of spectral reflectance from RGB images. Pattern Recognit. Image Anal..

[49] Nishidate I., Maeda T., Niizeki K., Aizu Y. (2013). Estimation of Melanin and Hemoglobin Using Spectral Reflectance Images Reconstructed from a Digital RGB Image by the Wiener Estimation Method. Sensors.

[50] Heikkinen V., Lenz R., Jetsu T., Parkkinen J., Hauta-Kasari M., Jääskeläinen T. (2008). Evaluation and unification of some methods for estimating reflectance spectra from RGB images. J. Opt. Soc. Am. A.

[51] Lapray P.J., Thomas J.B., Gouton P., Ruichek Y. (2017). Energy balance in Spectral Filter Array camera design. J. Eur. Opt. Soc.-Rapid Publ..

[52] Thomas J.B. Illuminant estimation from uncalibrated multispectral images. Proceedings of the 2015 Colour and Visual Computing Symposium (CVCS).

[53] Wang X., Thomas J.B., Hardeberg J.Y., Gouton P. (2014). Multispectral imaging: Narrow or wide band filters?. JAIC J. Int. Colour Assoc..

[54] Khan H.A., Thomas J.B., Hardeberg J.Y., Laligant O. (2017). Illuminant estimation in multispectral imaging. J. Opt. Soc. Am. A.

[55] Randeberg L.L., Winnem A., Blindheim S., Haugen O., Svaasand L. (2004). Optical classification of bruises. Proc. SPIE.

[56] Humphreys K., Ward T., Markham C. A CMOS camera-based pulse oximetry imaging system. Proceedings of the 2005 IEEE Engineering in Medicine and Biology 27th Annual Conference.

[57] Kong L., Yi D., Sprigle S., Wang F., Wang C., Liu F., Adibi A., Tummala R. (2010). Single sensor that outputs narrowband multispectral images. J. Biomed. Opt..

[58] Spigulis J., Oshina I., Berzina A., Bykov A. (2017). Smartphone snapshot mapping of skin chromophores under triple-wavelength laser illumination. J. Biomed. Opt..

[59] Bauer J.R., Bruins A.A., Hardeberg J.Y., Verdaasdonk R.M. (2019). A Spectral Filter Array Camera for Clinical Monitoring and Diagnosis: Proof of Concept for Skin Oxygenation Imaging. J. Imaging.

[60] Day D. (2003). Spectral Sensitivities of the Sinarback 54 Camera.

[61] Bauer J.R., Pedersen M., Hardeberg J.Y., Verdaasdonk R. (2017). Skin color simulation - review and analysis of available Monte Carlo-based photon transport simulation models. Color Imaging Conf..

[62] Jacques S. (1996). Origins of tissue optical properties in the UVA, visible, and NIR regions. Adv Opt Imaging Photon Migr..

[63] Jacques S.L., Prahl S.A. (2015). A Collaboration of Oregon Health & Science University, Portland State University, and the Oregon Institute of Technology. Optical Spectra. https://www.omlc.org.

[64] IEC (1999). International Standard: International Electrotechnical Commission.

